# Interleukin-12 as a Predictor for Unresponsiveness to the Hepatitis B Vaccine: A Novel Cut-Off Point in Children

**DOI:** 10.3390/children12101400

**Published:** 2025-10-17

**Authors:** Yudith Setiati Ermaya, Yunia Sribudiani, Reni Ghrahani, Quak Seng Hock, Dwi Prasetyo

**Affiliations:** 1 Department of Child Health, Faculty of Medicine Padjadjaran University, Dr. Hasan Sadikin General Hospital, Bandung 40161, Indonesia; reni.ghrahani@unpad.ac.id (R.G.); dwi.prasetyo@unpad.ac.id (D.P.); 2 Department of Basic Medical Sciences, Faculty of Medicine Padjadjaran University, Bandung 40161, Indonesia; y.sribudiani@unpad.ac.id; 3 Department of Paediatrics, National University Hospital, Singapore 119228, Singapore; senghock_quak@nuhs.edu.sg

**Keywords:** anti-HB, children, hepatitis B vaccine, interleukin-12, non-response

## Abstract

**Background**: Despite the widespread implementation of Hepatitis B vaccination, some children fail to develop protective immunity. Thus, identifying markers for vaccine unresponsiveness is crucial for optimizing current strategies. As interleukin-12 (IL-12), a central cytokine in Th 1 immune activation, has shown potential as a predictive biomarker, the aim of this study was to determine its role in the Hepatitis B vaccine response, highlighting novel findings regarding the threshold level of IL-12 in the pediatric population in doing so. **Methods**: A cross-sectional study was conducted on a community in Bandung City. The subjects were 7–12-month-old babies who had completed primary Hepatitis B vaccination (0, 2, 3, and 4 months of age). Blood tests for anti-HB examination were performed with a Chemiluminescent Microparticle Immunoassay (CMIA) to determine the response and non-response groups, and an Enzyme Immunosorbent Assay (ELISA) was used for IL-12 detection. Data were analyzed using the Kruskal–Wallis test, Chi-square test, Spearman Correlation, and Receiver Operating Characteristics. Statistical analysis was conducted with SPSS version 18.0. **Results**: The results of this study indicate that 4.5% of the subjects were unresponsive to the Hepatitis B vaccine. The most important finding was a significant correlation between IL-12 and the presence of anti-HB titers in the responsive and non-responsive groups (*p* = 0.016). The Receiver Operating Characteristics (ROC) curve for IL-12 identified a cut-off point of ≥10.65 pg/mL (>100 mIU/mL in anti-HBs), with a sensitivity of 64.23%, specificity of 68.75%, and accuracy of 65.2%. **Conclusions**: Interleukin-12 can be considered as an early candidate biomarker for responsiveness to the Hepatitis B vaccine in children.

## 1. Introduction

The Hepatitis B virus (HBV) continues to be a global health challenge, particularly in low- and middle-income countries. According to the World Health Organization (WHO), an estimated 254 million people were living with chronic Hepatitis B infection in 2022, with new infections affecting 1.2 million people each year. Estimations of the global HBV disease burden indicate that, by 2023, 253 million people will be living with HBV (a prevalence of 3.2%). Moreover, data indicate that 5.4 million of these will be aged 5 or younger, equivalent to a childhood prevalence of 0.7%; these figures include children unresponsive to the Hepatitis B (HB) vaccine.

Hepatitis B is most commonly spread from mother to baby at birth via perinatal transmission or exposure to infected blood (horizontal transmission) [1,2,3]. Hepatitis B can be prevented by administering the Hepatitis B vaccine, a safe and effective vaccine that provides protection against HBV infection to more than 95% of healthy infants, children, and young adults. Although the HBV vaccine is highly effective, nearly 5% of immunocompetent individuals fail to respond to the primary HBV series. Non-responsiveness in children is of particular concern due to their increased vulnerability to chronic HBV infection [4,5,6].

Vaccination has been a major breakthrough in the global effort to eradicate the virus and protect people from HBV infection [6,7,8,9]. Currently, HB vaccination is routinely recommended for infants, children, and adolescents. HB vaccination produces antibodies that provide protection, with the hepatitis B surface antibody (anti-HB) titer being ≥10 mIU/mL after completing the full primary vaccine series. In individuals who do not respond to the complete vaccine course and who are not protected from HBV infection, the WHO defines non-response as an anti-HB titer < 10 mIU/mL after three doses of the HB vaccine [1,2,3,4,5]. According to previous studies, non-responsiveness was found to be 4–10% [6,7].

Many studies have been conducted to examine factors contributing to non-responsiveness, from epidemiological factors to immunological mechanisms and to genetic polymorphisms involving cytokines, cytokine receptors, and antigen-presenting cells [10]. Immunogenetic factors and the cytokine response play crucial roles in vaccine efficiency. Interleukin-12 (IL-12) is a pro-inflammatory cytokine stemming from dendritic cells and macrophages, and it induces the differentiation of native clusters of differentiation (CD) 4+ T cells into T helper 1 cells, stimulating interferon-γ (IFN-γ) production. This response is essential for viral clearance and effective vaccine-induced immunity. As IL-12 regulates the development of adaptive immune cells, it plays a central role in coordinating innate immunity and determining the type and duration of adaptive immune responses [11,12]. Research previously conducted in Egypt showed a correlation between IL-12 and anti-HBs as a response to the Hepatitis B vaccine [13]. However, as of this study, there is no IL-12 level or cut-off point that indicates responsiveness to the HB vaccine, especially in children.

The aim of our study, therefore, was to determine the role of IL-12 in the Hepatitis B vaccine response, highlighting new findings regarding the cut-off point for IL-12 levels in determining responsiveness to the HB vaccine in the pediatric population.

## 2. Materials and Methods

### 2.1. Participants and Procedure

This study was a cross-sectional study conducted on a community in Bandung City. Participants were selected according to the following inclusion criteria: infants aged 7–12 months, clinically healthy, and having received the complete primary HB vaccination series (4 doses at ages 0, 2, 3, and 4). The sample size was determined by samples taken consecutively between October 2023 and August 2024. Blood samples were collected from subject and measuring the hepatitis B surface antibody (anti-HBs) and Interleukin-12 (IL-12). Anti-HB examinations were carried out to determine the responsive and non-responsive groups, and IL-12 detection was performed based on the levels of responsiveness in the groups.

### 2.2. Serum Sampling

**Figure 1:**
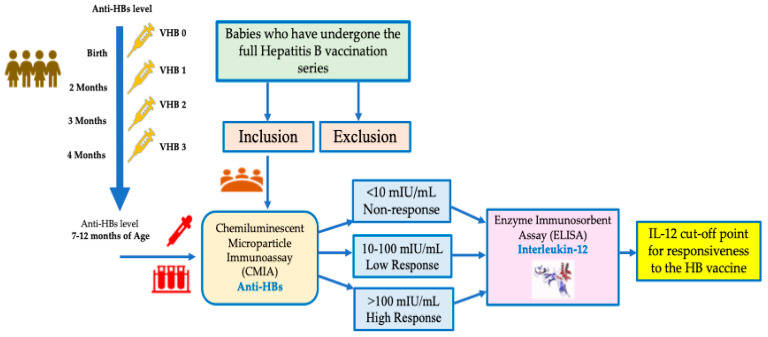
Study flow chart.

The laboratory procedure for measuring anti-HBs involved direct microparticle antibody capture using the Chemiluminescent Microparticle Immunoassay (CMIA) method, used to measure the amount of anti-HBs in human serum and plasma. An antigen–antibody complex forms if anti-HBs are present in the sample, with a test range of 3.1–1000 mIU/mL (criteria comprise non-response (<10 mIU/mL), low response (10–100 mIU/mL), and high response (>100 mIU/mL) [4,5,14]. The IL-12 examination uses an Enzyme Immunosorbent Assay (ELISA) with Elabscience^R^, this kit recognize human IL-12 in samples, with sensitivity 9.38 pg/mL and detection range 15.63–1000 pg/mL. (Figure 1) [15].

**Figure 2:**
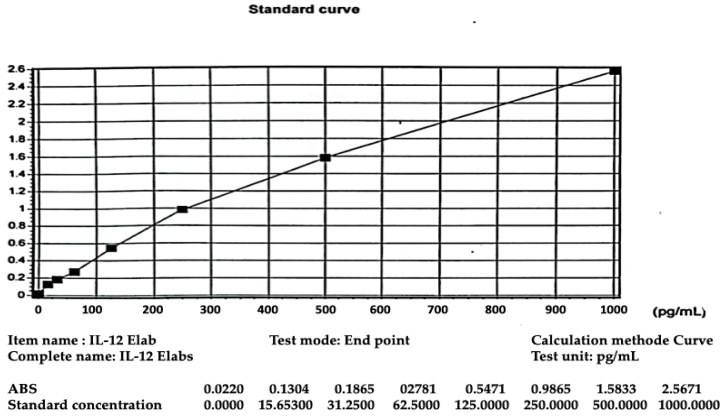
Standard curve IL-12 levels.

For quality control IL-12 levels in this study we used standard curves, as shown in Figure 2.

### 2.3. Statistical Analysis

Statistical analysis was conducted with Statistical Package for Social Sciences (SPSS) version 18.0 (SPSS Inc., Chicago, IL, USA). The differences between IL-12 levels in the responsive groups (non-response, low response, and high response) were analyzed using the Kruskal–Wallis test, and Spearman Correlation was performed to analyze the correlation between IL-12 and the anti-HB titer levels in the groups. Additionally, a cut-off point was calculated, a Receiver Operating Characteristic (ROC) curve was established, and the magnitude, sensitivity, and specificity were calculated. A *p*-value less than 0.05 was considered statistically significant. Results are displayed in tables.

## 3. Results

### 3.1. Subject Characteristics

This study included 155 subject 7–12-month-old babies. The laboratory results of the anti-HBs measured were used for grouping responsiveness to HB vaccination and IL-12 levels. Table 1 presents the characteristics of the study’s subjects, including their sex, age, and laboratory values.

The most important findings from this study were that there were seven (4.5%) infants who were unresponsive to HB vaccination, the sex distribution (50.3% girls vs. 49.7% boys) was nearly equal, and the age of completing the primary HB vaccine series was less than 6 months (59.4%), in accordance with the national HB vaccination program, with vaccines administered at the ages of 0, 2, 3, and 4 months. Table 1.

### 3.2. Results and Analysis

As can be seen in Table 2, the mean age is 9.7 months from a range of 7–12 months, the average anti-HB titer is 407.85 mIU/mL, and the mean interleukin-12 level is 86.88 pg/mL. From the results of the data normality test for the three variables studied, a *p* value of <0.05 was obtained, meaning that the data was not normally distributed. Thus, in order to analyze the correlation between the variables, Spearman Rank Correlation was used, as indicated in Table 3.

**Figure 3:**
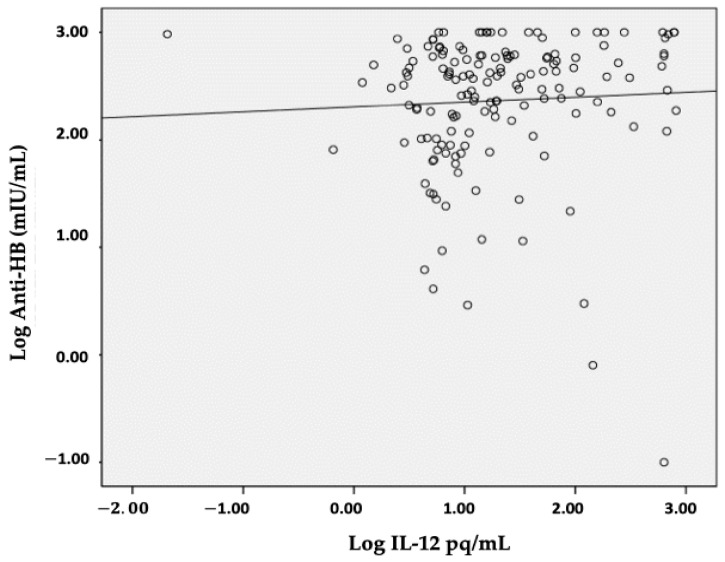
Correlation between IL-12 levels and anti-HB titers in the responsiveness to HB vaccination. (r = 0.193; *p* = 0.016).

In this study, the laboratory results of anti-HB measurements were used for grouping responsiveness to HB vaccination and IL-12 levels. As shown in Figure 3, a positive correlation was obtained, indicating that high IL-12 levels were in line with high anti-HB levels and that, as a consequence, high IL-12 levels were positively correlated with better rates of responsiveness to the Hepatitis B vaccine.

As shown in Table 3, there is a significant difference in IL-12 levels based on vaccine responsiveness, as indicated by the anti-HB titer (*p* = 0.013) and IL-12 levels in the non-response vs. high response groups (10.65 (4.34–634.26) pg/mL vs. 17.26 (0.02–814.77) pg/mL), demonstrating a difference in distribution based on responsiveness to the HB vaccine, with a correlation of r = 0.193 and *p* = 0.016. Even though the correlation is weak, it is significant.

A comparative analysis based on the different responsive groups was performed using the Mann–Whitney test, resulting in a comparison of the NR vs. LR (*p* = 0.034), NR vs. HR (*p* = 0.955), and LR vs. HR (*p* = 0.003) groups. From these data, it was found that anti-HB levels of the HR group (or >100 mIU/mL) showed significant differences, indicating a good immune response according to the IL-12 levels. The IL-12 levels in this study ranged widely, with this variability likely caused by several factors, including biological differences, clinical conditions, and underlying or subclinical conditions that could have affected immune activation.

The ROC curve results for the IL-12 and anti-HB levels (in the LR and HR groups) were used to discriminate between non-responsiveness and responsiveness via Area Under the Curve (AUC) analysis. We found that an anti-HB response >100 mIU/mL, with cut-off levels > 10.65 pg/mL for IL-12 and an AUROC = 0.649 (CI 95%; 0.565–0.724), indicates a moderate discriminative ability, with *p* values = 0.004 showing statistically significant differences in IL-12 levels between the LR and HR groups (Table 4). Clinically, IL-12 may serve as a risk indicator for vaccine non-responsiveness, but it is not yet strong enough to be used as a sole predictor. Its primary role is more in early development and in combination with other factors.

**Figure 4:**
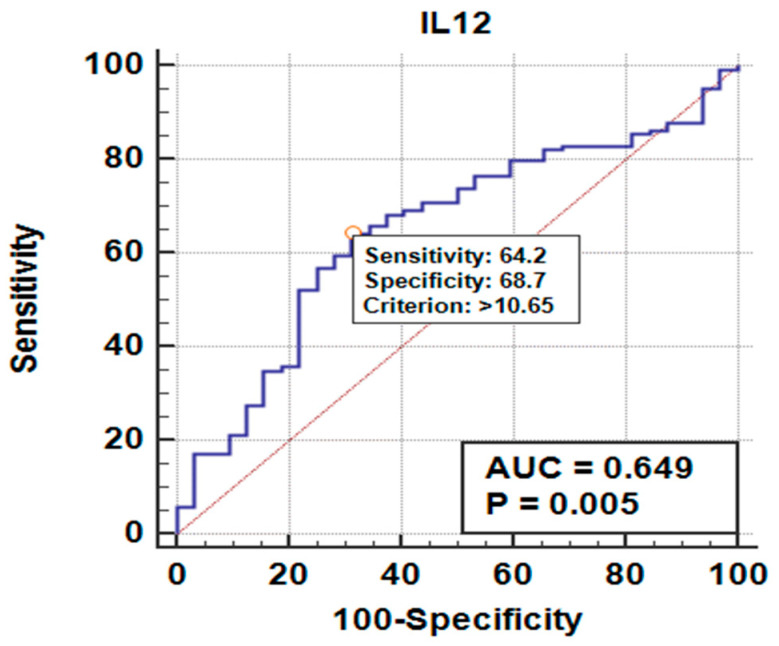
A histogram of ROC curve analysis. Receiver Operating Characteristics Curve to determine the cut-off value of IL-12 in predicting the response to HR with anti-HBs (>100 mIU/mL). Cut-off value > 10.65 pg/mL. Sensitivity = 64.23%, Specificity = 68.75%, PPV = 88.8%, NPV = 33.3%, Accuracy = 65.2%.

To demonstrate this point, an assessment was performed using a table and ROC curve, as shown in Figure 4.

Figure 4 depicts the cut-off value for IL-12 levels based on calculations using the Receiver Operating Characteristic (ROC) model. For IL-12 levels, the cut-off value was >10.65 pg/mL with a *p*-value of 0.005, resulting in a sensitivity of 64.23%, a specificity of 68.75%, a positive predictive value (PPV) of 88.8%, a negative predictive value (NPV) of 33.3%, and an accuracy of 65.2%. These values indicate statistical significance and a moderately strong discriminative ability of IL-12 as a biomarker; from these findings, IL-12 can be used as a predictor of the anti-HB response (Table 4).

The following Table 5 depicts the relationship between cut-off IL-12 values and anti-HB occurrence, with the IL-12 cut-off point being >10.65 pg/mL.

Table 5 depicts the cut-off values, indicating that those with IL-12 levels > 10.65 pg/mL have a 3.95-fold increased risk of anti-HB being >100 mIU/mL compared to those with IL-12 levels ≤ 10.65 pg/mL. The implication of this for the community is that if a child has an anti-HB titer of more than >100 mIU/mL, the child has a good immune status, with a healthy immune response.

## 4. Discussion

The most important finding we observed was that 4.5% of the participants were non-responsive to HB vaccination, a finding very similar to that of other studies, in which it was found that 4–10% of people globally are non-responsive to HB vaccination [1,16]. Moreover, the sex distribution in our study (50.3% girls; 49.7% boys) was nearly equal and similar to that of a previous study conducted in Egypt [17].

Age is known to be a factor in the strength and duration of the immune response, with younger children generally having a stronger and longer-lasting response [18]. In this study, the age group was 9–12 months (74.8%), but the analysis showed no significant differences between non-responsive and responsive babies. Thus, age characteristics in the non-responsive group were not statistically significant in this study. This aligns with findings from another study conducted in Uganda [19].

Interleukin-12 is a cytokine that plays an important role in the immune system response, as it can induce IFN-γ and increase the development of the immune response through Th1 [20,21,22]. In this study, higher (and significantly different) interleukin-12 levels were found in the responsive group compared to the non-responsive group (10.65 (4.34–634.26) pg/mL vs. 17.26 (0.02–814.77) pg/mL), and a low correlation was found between anti-HBs and IL-12, in line with research previously conducted in Egypt [13]. Although the correlation between IL-12 levels and anti-HB titers was weak (r = 0.193), this modest association may still hold clinical relevance. Even small changes in IL-12 levels could indicate underlying immune responsiveness, especially in populations at risk of non-responsiveness. However, the weakness of the association suggests that IL-12 alone is unlikely to serve as a definitive predictor and should instead be considered in combination with other biomarkers or host factors. Several studies on IL-12 deficiency and unresponsiveness in children have shown that unresponsive children exhibit significantly lower IL-12 levels after HBsAg stimulation compared to responsive children [10]. These lower cytokine levels are accompanied by inadequate IFN-γ production and inadequate B cell support, resulting in low or undetectable anti-HB titers. Moreover, studies have highlighted a correlation between IL-12 concentrations and vaccine response, suggesting that below the threshold level of ≤10.65 pg/mL, the immune response may be inadequate [13,23,24].

In this study, a positive correlation was found between IL-12 levels and post-vaccination Hepatitis B antibody levels. The responsive group had significantly higher IL-12 levels, and there was a positive correlation between IL-12 levels and anti-HB titers. Moreover, interleukin-12 levels were higher in the responsive group. However, in both the non-responsive and responsive groups, there was a positive correlation between IL-12 levels and anti-HB levels, indicating a better response [13,25,26].

We found that an anti-HB response >100 mIU/mL with an IL-12 level cut-off point > 10.65 pg/mL showed a moderate discriminatory ability, with statistically significant differences found in the IL-12 levels between the LR and HR groups. This shows that, in order to develop responsiveness to the HB vaccine, anti-HBs > 100 mIU/mL and IL-12 levels > 10.65 pg/mL are required to obtain protection against Hepatitis B for 20 years or possibly for life [1].

Interleukin-12 may have potential as an early biomarker for identifying children at risk of non-responsiveness to HB vaccination. However, this study is limited by the weak correlation and moderate discriminative ability observed, in addition to its relatively small sample size, single-center design, and variability in participant age (months), which may affect the generalizability of the findings. Therefore, IL-12 should only be considered a preliminary candidate biomarker, requiring validation in larger, multi-center studies. Ongoing research into IL-12 threshold levels will offer new insights into pediatric vaccine monitoring. Furthermore, the implementation of IL-12 threshold-based or cut-off levels for screening may enable early interventions such as booster doses or adjuvant therapy for children at risk of unresponsiveness. Future large-scale, multicenter studies are needed to validate these thresholds and integrate IL-12 testing into routine immunization programs.

In summary, further research is necessary to validate this biomarker across diverse populations and clinical settings.

## 5. Conclusions

This study highlights the utility of inteurleukin-12 in predicting non-responsiveness to the Hepatitis B vaccine, indicating that IL-12 can be an initial biomarker for examining responsiveness. Nevertheless, IL-12 should be considered a candidate biomarker rather than a definitive predictor. Moreover, anti-HB titers > 100 mIU/mL and IL-12 levels > 10.65 pg/mL are also required to obtain a good response.

## Figures and Tables

**Table 1:** Subject characteristics.

Characteristics	n = 155(%)
Sex	
Boy	77 (49.7)
Girl	78 (50.3)
Age (months)	
7–<9	39 (25.2)
9–12	116 (74.8)
The age at which the primary Hepatitis B vaccination series was completed (months)	
≤6	92 (59.4)
>6	63 (40.6)
Anti-HB titer (mIU/mL)	
<10 (NR)	7 (4.5)
10–100 (LR)	25 (16.1)
>100 (HR)	123 (79.4)

Note: NR: non-response; LR: low response; HR: high response. Anti-HBs: hepatitis B surface antibody.

**Table 2:** Descriptive statistics of age, anti-HB titers, and IL-12 levels in children who received Hepatitis B vaccination.

Variable	Statistical Measures				Data Normality Test (*p* * Value)
	Mean	SD	Median	Range	
Age (months)	9.7	1.64	10.0	7–12	<0.001
Anti-HBs (mIU/mL)	407.85	314.88	370.60	0.1–1000	0.001
IL-12 (pg/mL)	86.88	183.84	14.33	0.02–814.78	<0.001

Note: (*) Kolmogorov–Smirnov test.

**Table 3:** Correlation of IL-12 levels with the anti-HB titer response.

Variable	Anti-HB Titer (mIU/mL)			*p* * Value	r	*p* ** Value
	<10 (NR) n = 7	10–100 (LR) n = 25	>100 (HR) n = 123			
IL-12 (pg/mL)	10.65 (4.34–634.26)	7.42 (0.65–90.09)	17.26 (0.02–814.77)	0.013	0.193	0.016

Note: (*) Kruskal–Wallis test; (**) Spearman Rank Correlation. NR: non-response; LR: low response; HR: high response.

**Table 4:** Cut-off values for IL-12 levels as a predictor of the response to anti-HBs.

Anti-HB Response	Cut-Off IL-12 Level	AUROC (CI 95%)	*p* Value
>10 mIU/mL (LR)	≤110.39 pg/mL	0.516 (0.435–0.597)	0.906
>100 mIU/mL (HR)	>10.65 pg/mL	0.649 (0.565–0.724)	0.004

Note: AUROC: Area Under the Receiver Operating Characteristic Curve; CI: confidence interval. IL: interleukin; LR: low response, HR: high response.

**Table 5:** Relationship between cut-off IL-12 values and anti-HB occurrence.

Cut-Off Value	Anti-HBs (mIU/mL)		*p* * Value	OR (CI 95%)
	>100	≤100		
IL-12 (pg/mL):			0.001	
>10.65	79	10		3.95 (1.72–9.09)
≤10.65	44	22		

(*) Chi-square test; OR (95% CI): odds ratio with a 95% confidence interval.

## Data Availability

Data availability is restricted due to privacy concerns or ethical constraints. Please contact the corresponding author for further information.

## Abbreviations

The following abbreviations are used in this manuscript:

AUROC	Area Under the Receiver Operating Characteristics Curve
CD	Cluster of differentiation
CMIA	Chemiluminescent Microparticle Immunoassay
HB	Hepatitis B
HBV	Hepatitis B virus
HR	High Response
IL-12	Interleukin-12
LR	Low Response
NPV	Negative predictive value
NR	Non-Response
OR	Odd Ratio
PPV	positive predictive value
ROC	Receiver Operating Characteristics
WHO	World Health Organization
